# First occurrence of *Corynespora cassiicola* infecting chia plant in Bangladesh and sensitivity of the pathogen to selected fungicides

**DOI:** 10.1371/journal.pone.0349471

**Published:** 2026-09-17

**Authors:** Avi Kumar Badhon, Dipali Rani Gupta, Swapan Kumar Paul, Julfikar Ali, Md. Moshiur Rahman, Tofazzal Islam

**Affiliations:** 1 Institute of Biotechnology and Genetic Engineering, Gazipur Agricultural University, Gazipur, Bangladesh; 2 Department of Agronomy, Bangladesh Agricultural University, Mymensingh, Bangladesh; University of Duhok, IRAQ

## Abstract

Chia (*Salvia hispanica* L.) is an emerging crop in Bangladesh valued for its medicinal properties and economic significance. In March 2024, target spot-like symptoms were observed in an experimental chia field (24.75° N, 90.50° E) at Bangladesh Agricultural University in Mymensingh, Bangladesh, with disease incidence ranged from 23% to 47% across approximately 0.25 ha. Initially appearing as brick-red spots, these symptoms developed into target-shaped concentric rings, affecting leaves, stems, and inflorescences. A total of 24 fungal isolates were recovered from infected tissue; two representative isolates (BGECh-3 and BGECh-4) were randomly selected for detailed characterization. Pathogen identity was established through morphological traits, multilocus phylogenetic analysis of internal transcribed spacer (ITS) and elongation factor 1-alpha (*TEF-1α*) gene sequences identifying the causal agent as *Corynespora cassiicola.* Pathogenicity was confirmation through Koch’s postulates, and the isolates demonstrated a broad host range and were pathogenic to brinjal, chili, bottle gourd, country bean, tomato, and soybean. In vitro fungicide sensitivity assays with seven commercial fungicides showed that both isolates were highly sensitive to Goldazim (50% carbendazim), which caused 98.05 ± 2.88% mycelial growth inhibition at 10 µg mL ^−^ ¹ and complete inhibition at higher concentrations, with the lowest EC_50_ (0.76 µg mL ^−^ ¹). Conza (10% Hexaconazole) and Amistar top (18.2% azoxystrobin + 11.4% difenoconazole) also exhibited high efficacy, achieving 86.17 ± 1.31% and 67.77 ± 1.34% inhibition, respectively, at the same concentration, with EC_50_ values of 1.34 and 1.09 µg mL ^−^ ¹, respectively. Tilt (25% propiconazole) showed moderate activity (59.30 ± 2.08% inhibition at 10 µg mL ^−^ ¹; EC_50_ = 4.75 µg mL ^−^ ¹), whereas the remaining fungicides exhibited comparatively lower efficacy and did not produce reliable EC_50_ estimates. This study represents the first report of target spot disease of chia caused by *C. cassiicola* in Bangladesh and provides insights for effective disease management strategies.

## Introduction

Chia (*Salvia hispanica* L.), a newly introduced seed crop in Bangladesh, belongs to the family Lamiaceae and has gained considerable attention due to its exceptional nutritional and functional properties [1]. Chia seeds are rich in omega-3 fatty acid, high-quality protein, vitamins, antioxidants, and dietary fiber [2,3], making them highly desirable for the functional food, nutraceutical, and pharmaceutical industries. Beyond its nutritional benefits, chia is well adapted to diverse agroecological conditions and offers farmers an attractive alternative crop for diversifying production systems and enhancing farm profitability. Consequently, the sustainable cultivation of chia has considerable agronomic importance, particularly in countries like Bangladesh seeking to expand high-value crops while improving food and nutritional security [2,3].

Emerging fungal diseases are becoming an increasing threat to newly introduced and expanding crops as climate change alters pathogen distribution, enhances survival under previously unsuitable environmental conditions, and facilitates dispersal into new production areas [4–9]. Broad-host-range pathogens are of particular concern because they can readily colonize novel hosts, increasing the risk of disease emergence as agricultural diversification expands.Such host jumps and long-distance dispersal have been documented in major pathogens, including *Rhizoctonia solani* and *Puccinia graminis* f. sp. *tritici* [10]. Climate change in Bangladesh has significantly impacted crop production and altered disease dynamics by increasing environmental stress on plants and creating favorable conditions for the emergence and spread of plant pathogens. The outbreak of wheat blast in 2016, caused by the fungus *Magnaporthe oryzae* pathotype *Triticum*, is considered a consequence of changing weather patterns and the possible introduction of contaminated seed from abroad [8,11]. Although this pathogen is largely host-specific, which has so far limited its spread to other agriculturally important crops, the situation could become devastating for broad-host-range pathogens, such as *Corynespora cassiicola*, are inherently capable of infecting diverse plant species, making the emergence of target spot disease on newly introduced crops such as chia a plausible consequence of ongoing environmental change and pathogen dispersal.

*C. cassiicola* is a highly polyphagous fungal pathogen reported in more than 70 countries and infecting over 400 plant species, including economically important food, fiber, and horticultural crops [12]. This destructive necrotrophic fungus can infect multiple plant organs, causing characteristic necrotic lesions, premature defoliation, and substantial yield losses [13,14]. The pathogen displays considerable biological and pathogenic variability; isolates often differ markedly in growth rate, sporulation capacity, temperature optima, and virulence, even within similar ecological zones [15,16]. Such variability underscores the importance of accurate pathogen identification and thorough biological characterization, particularly when the pathogen is newly detected on a crop in a specific country. Although *C. cassiicola* has been widely reported worldwide, its occurrence on chia has not yet been documented in Bangladesh. Therefore, a comprehensive understanding of the pathogen associated with target spot of chia in Bangladesh is essential for elucidating disease epidemiology, tracing possible routes of introduction, assessing future outbreak risks, and developing effective management strategies.

The control of fungal diseases relies predominantly on the application of synthetic fungicides. *C. cassiicola*

Fungicides belonging to several chemical groups, including succinate dehydrogenase inhibitors (SDHIs), quinone outside inhibitors (QoIs), sterol demethylase inhibitors (DMIs), and benzimidazoles, have been widely used to manage *C. cassiicola* infections in susceptible crops [17–19]. However, fungicide resistance in *C. cassiicola* populations have been widely documented against several of these fungicide classes, highlighting the importance of monitoring fungicide sensitivity and implementing appropriate resistance management strategies [17,20,21]

In this study, typical target spot–infected chia samples were collected in March 2024 from an experimental field at Bangladesh Agricultural University. The associated fungal isolates were obtained and their pathogenicity was confirmed following Koch’s postulates. Accurate identification of the pathogen was accomplished through detailed morphological characterization combined with multigene phylogenetic analysis. Furthermore, the in vitro sensitivity of selected fungicides was assessed to determine the most effective compounds against the pathogen. The findings of this study provide important insights for developing effective management strategies for target spot disease in Bangladesh.

## Materials and Methods

### Collection and isolation of fungal isolates

The suspected target spot diseased plants were collected from an experimental field of BAU, Mymensingh. Tissue pieces (5 mm × 5 mm) were collected from the lesion-healthy tissue interface of infected leaves, stems, and inflorescences, followed by surface sterilization with 75% ethanol for 1 minute, rinsing three times with sterile water, and drying on sterile absorbent paper. Infected tissues were placed onto PDA medium and incubated at 25 °C under a 12 h light/12 h dark photoperiod for 2–3 days for fungal growth. Emerging fungal colonies were initially differentiated based on distinct colony morphology, including colony color, texture, growth pattern, and margin morphology. Representative colonies were purified using the single-spore technique on potato dextrose agar (PDA) at 25 °C in the dark. The isolates were stored at −80 °C for further use.

### Morphological characterization

Morphological characteristics, including colony size, shape, color, and growth rate, were assessed by culturing the isolates on (PDA). Briefly, a 5 mm mycelial plug taken from the actively growing margin of a colony was placed at the center of a PDA plate and incubated at 25 °C in the dark for 10 days. Colony diameter was measured after 3, 7, and 10 days of incubation using a ruler and the growth rate (cm day ^−^ ¹) was calculated. Conidia were collected using the whole-mycelium harvest method described by Zhao [22], in which the entire fungal colony was flooded with 10 mL of sterile distilled water with 0.1% (v/v) Tween 20 and gently brushed with a sterile soft brush to dislodge the conidia into suspension. The resulting suspension was filtered through two layers of sterile gauze to remove mycelial debris. The size, shape, and arrangement of conidia and conidiophores were examined under a light microscope (40 × magnification) (LEICA DM 1000 Fluorescence microscope), and images were captured using an attached (LEICA MC 190 HD) microscope camera.

### Molecular characterization

Genomic DNA was extracted from two randomly selected fungal isolates (BGECh-3 and BGECh-4) by scraping mycelia from 7-day-old cultures using the Wizard® Genomic DNA Purification Kit (Promega Corporation, Madison, WI, USA), following the manufacturer’s instructions. DNA quality and concentration were assessed using a Qubit™ 4 Fluorometer (Thermo Fisher Scientific). The internal transcribed spacer (ITS) region of the ribosomal DNA and the translation elongation factor 1-alpha (*TEF-1α*) gene were amplified for species-level identification. PCR amplification was performed using the primer pairs ITS1/ITS4 for ITS region [23] and EF595F/EF1160R for (*TEF-1α*) gene, following established protocols [25]. The primer sequences and expected amplicon sizes are provided in Table 1. Each 50 μl PCR reaction volume containing 5 μl of gDNA (50 ng/μL), 2.5 μl of each forward and reverse primers (10 pmol/μL), 25 μl of 2x GoTaq G2 green PCR MasterMix (Promega^TM^, Madison, WI, USA), and 15 μl of nuclease-free water (Promega^TM^, Madison, WI, USA). For ITS amplification, thermal cycling conditions consisted of an initial denaturation at 95 °C for 2 min; followed by 35 cycles of 95 °C for 40 s, 55 °C for 40 s, and 72 °C for 60 s; and a final extension at 72 °C for 5 min. For *TEF-1α* amplification, the program included an initial denaturation at 95 °C for 2 min; followed by 35 cycles of 95 °C for 40 s, 54 °C for 35 s, and 72 °C for 60 s; and a final extension at 72 °C for 5 min. The amplified PCR products were visualized under a UV transilluminator, purified, and sent for sequencing to Apical Scientific Sequencing. The obtained sequences were compared with reference ITS and *TEF-1α* sequences of *C. cassiicola* available in the NCBI GenBank database using the BLAST algorithm. Multiple sequence alignment and homology analyses were performed using MAFFT version 7 [26]. The newly generated sequences were deposited in GenBank. Phylogenetic relationships were inferred using the Maximum likelihood method with the General Time Reversible (GTR + G + I) substitution model implemented in MEGA version 11, based on 1,000 bootstrap replicates [27].

**Table 1:** Primer used for PCR amplification and DNA sequencing for molecular identification of fungal isolates.

Primer name	Primer sequence (5’ – 3’)	Amplicon size (bp)	Target region/Gene	Reference
ITS1 ITS4	TCCGTAGGTGAACCTGCGG TCCTCCGCTTATTGATATGC	500	Internal transcribed spacer regions 1 and 2 (ITS1, ITS2)	[23]
EF1-728F EF1-986R	CATCGAGAAGTTCGAGAAGG TACTTGAAGGAACCCTTACC	400	Translation elongation factor1- α (*tef1*)	[25]

### Inoculum preparation and pathogenicity assay

For the preparation of fungal inoculum, the isolates were cultured on potato dextrose agar (PDA) at 25 °C in the dark for 10 days. The mycelial mats were then flooded with 15 mL of sterile distilled water containing 0.1% Tween 20 and gently brushed with a sterile soft brush to release the conidia. The resulting suspension was filtered through two layers of sterile gauze into 50 mL Falcon tubes to remove mycelial debris. The conidial concentration was adjusted to 1 × 10^5^ spores/mL using a hemocytometer prior to inoculation.

For pathogenicity assays, 1 mL of conidial suspension (1 × 10^5^ spores/mL) from each of the two *C. cassiicola* isolates was sprayed onto one-month-old chia seedlings. Each isolate was applied to three independent plants per experiment, and the pathogenicity assay was independently repeated six times using separate inoculation events to ensure reproducibility. Additionally, inoculations were performed on stems and inflorescences. Control plants were sprayed with sterile distilled water containing 0.1% Tween 20. After inoculation, plants were maintained in a growth chamber at 25 ± 2 °C under a 16 h light/8 h dark photoperiod and high relative humidity (>90%) for 48 h to facilitate infection. Symptom development was monitored at every two-day interval. To fulfill Koch’s postulates, the pathogen was re-isolatedfrom symptomatic tissues of artificially inoculated plants, purified on PDA plates and confirmed by comparison with the original isolate based on morphological characteristics.

To assess the host range of *C. cassiicola,* isolates BGECh-3 and BGECh-4 were inoculated onto representative vegetable crops commonly cultivated in Bangladesh, including tomato (cv. BARI Tomato-5), chili (cv. KARASHI 5424), brinjal (cv. BARI Begun-6), soybean (cv. BARI Soybean-5), country bean (cv. BARI Seam-1), and bottle gourd (cv. BARI Lau-1). Seedlings were grown in plastic pots containing potting mix, and 20–30 days aged plants were inoculated with the pathogen following the procedure described above. Each treatment included appropriate controls, and symptom development was monitored under controlled conditions.

### Fungicides sensitivity assay

The sensitivities of *C. cassiicola* isolates to seven fungicides were assessed by determination of their effective concentration using poisonplate assays as described by [28,29]. The fungicides tested include carbendazim (Goldazim ® 500 SC, Square Pharmaceuticals PLC, Bangladesh), mancozeb (Ticozeb ® 80 WP, Square Pharmaceuticals PLC, Bangladesh), hexaconazole (Conza Plus 10 SC, ACI crop Care), propiconazole (Tilt 250 EC, Syngenta), 50% Tebuconazole combine with 25% Trifloxystrobin (Nativo 75 WG, Bayer Crop Science), combination of 18.2% Azoxystrobin and 11.4% Difenoconazole (Amistar top 325 SC) and 37.5% Carboxin combined with 37.5% Thiram (Provax 200 WP, Hossain Enterprise C.C. Limited).

Stock solution (1000 μg ml^−1^) of each fungicide was prepared in sterile distilled water and incorporated into autoclaved PDA (pH 7.0) medium after cooling to 50–55°C to obtain final concentrations 0.1, 1, 10, 50, and 100 μg ml^−1^. Fungicide-amended media were poured into Petri dishes, while non-amended PDA prepared with sterile distilled water served as the control. Subsequently, a 5-mm mycelial plug was taken from actively growing edge of a 7-day-old culture, placed at the center of each plate, and incubated in the dark at 28°C for 10 days.

Colony diameter (minus the original diameter of the inoculation plug) was measured along two perpendicular axes, and mean value was used to calculate radial growth according to [30]. Relative growth inhibition (RGI) was calculated according to the following formula:


$$\begin{array}{c} RGI\%=\left[\frac{\left(D\;contrl-D\;treatment\right)}{D\;control}\right]\times \;\text{1}\text{0}\text{0}, \end{array}$$

where D = diameter of the colony. Each isolate was tested for 6 replicates and the experiment was repeated twice independently.

The effective concentration to inhibit 50% of mycelial growth (EC_50_) was estimated by fitting a four-parameter log-logistic dose-response model using the ‘drc’ package (version 3.0−1) in R software (version 4.6.1) [31]. EC_50_ values were derived from the fitted curves based on relative growth inhibition data, and model parameters were used to describe the dose-response relationship for each fungicide.

### Statistical analysis

All experiments were conducted using a completely randomized design (CRD) with six replicates per treatment. Data were analyzed using the R software (version 4.6.1). Data on disease incidence, disease severity, and mycelial growth inhibition were analyzed using analysis of variance (ANOVA) to determine significant differences among treatments. Mean comparisons were performed using Fisher’s least significant difference (LSD) test at *P* ≤ 0.05, and results are presented as mean ± standard error (SE). The effective concentration required to inhibit 50% of mycelial growth (EC_50_) was estimated by fitting a four-parameter log-logistic dose-response model using the drc package in R, and 95% confidence intervals were calculated for each estimate.

## Results

### Disease symptoms and severity

**Fig 1:**
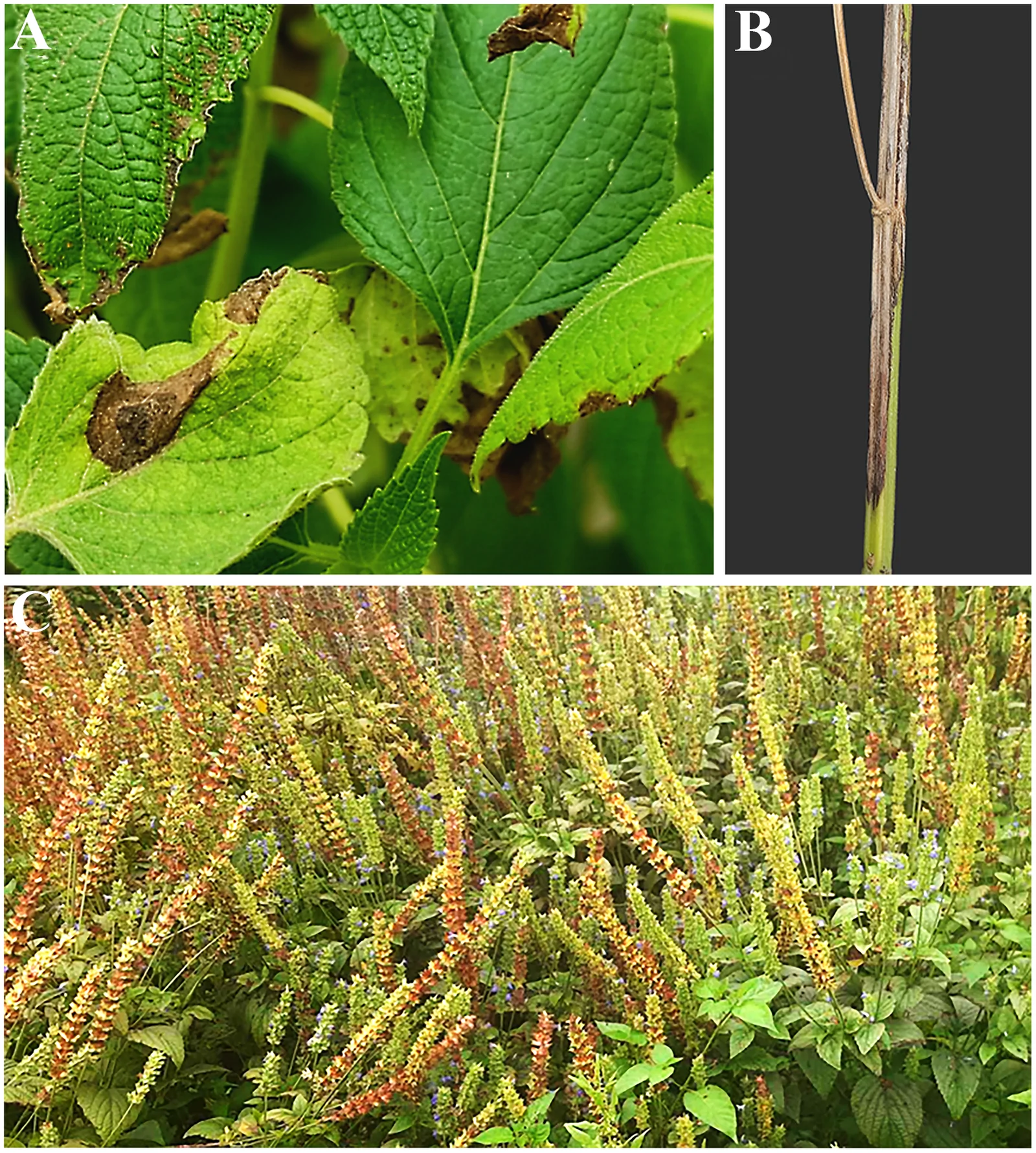
Symptoms of target spot disease caused by *C. cassiicola* on chia under field conditions. (A) necrotic, circular to irregular lesions with concentric rings on leaves; (B) elongated dark lesion on stems; and (C) infected inflorescences showing blight symptoms in a naturally infected field.

In March 2024, distinctive target spot symptoms were observed in an experimental chia field (24.75° N, 90.50° E) at Bangladesh Agricultural University in Mymensingh, Bangladesh. The disease, initially detected during the vegetative stage (approximately 3 weeks after sowing), worsened significantly during the flowering stage (approximately 8 weeks after sowing). The initial symptoms manifested as brick-red spots (Fig 1A). These symptoms developed into target-shaped concentric rings, affecting leaves, stems, and inflorescences (Fig 1B,C), indicating the ability of the pathogen to infect multiple aerial parts of the host. Approximately 0.25 hectares of chia field was affected with disease incidence ranging from 23% to 47%. A total of 24 fungal isolates were collected from eight infected chia plants, with at least three from each sample.

### Morphological characterization

**Fig 2:**
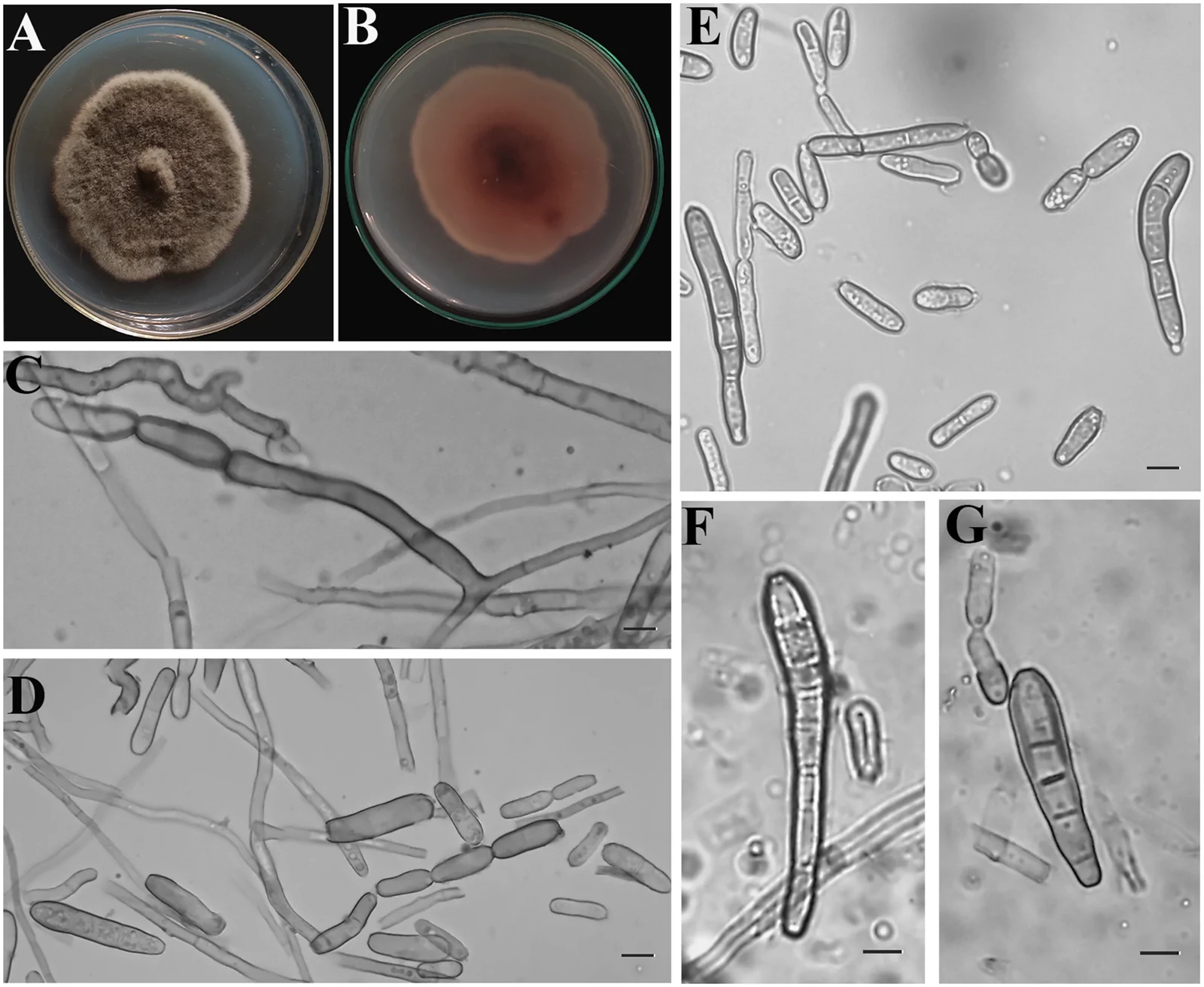
Morphological characteristics of *C. cassiicola* isolates BGECh-3 culture on PDA medium collected from diseased chia plant. (A) colony morphology (observed view); (B) reverse view; (C) conidia with conidiophore; (D) chain-like conidial arrangement; (E) conidia with various sizes and shapes; (F–G) obclavate-shaped conidia.

Fungal colonies on potato dextrose agar (PDA) were initially olivaceous, gradually turning brown with age, and exhibited mean radial growth rate of 0.8 ± 0.05 cm day^-1^(Fig 2A,B). The conidiophores were straight to slightly curved, smooth, unbranched, and grayish-brown in color (Fig 2C). No significant variation in conidial size or shape was observed among the isolates examined. Conidia were produced either singly or in chains on the conidiophores (Fig 2C,D). Although different conidial shapes were observed, straight forms predominated over obclavate or cylindrical types (Fig 2E). The conidia were obclavate to cylindrical in shape and contained 2–10 pseudosepta, most commonly 2–5. Measurements of 100 conidia revealed a size range of 9–130 μm in length and 5–12 μm in width (Fig 2D–G). Based on these morphological characteristics, the isolates were identified as *Corynespora* sp [32].

### Molecular identification of fungal isolates

**Fig 3:**
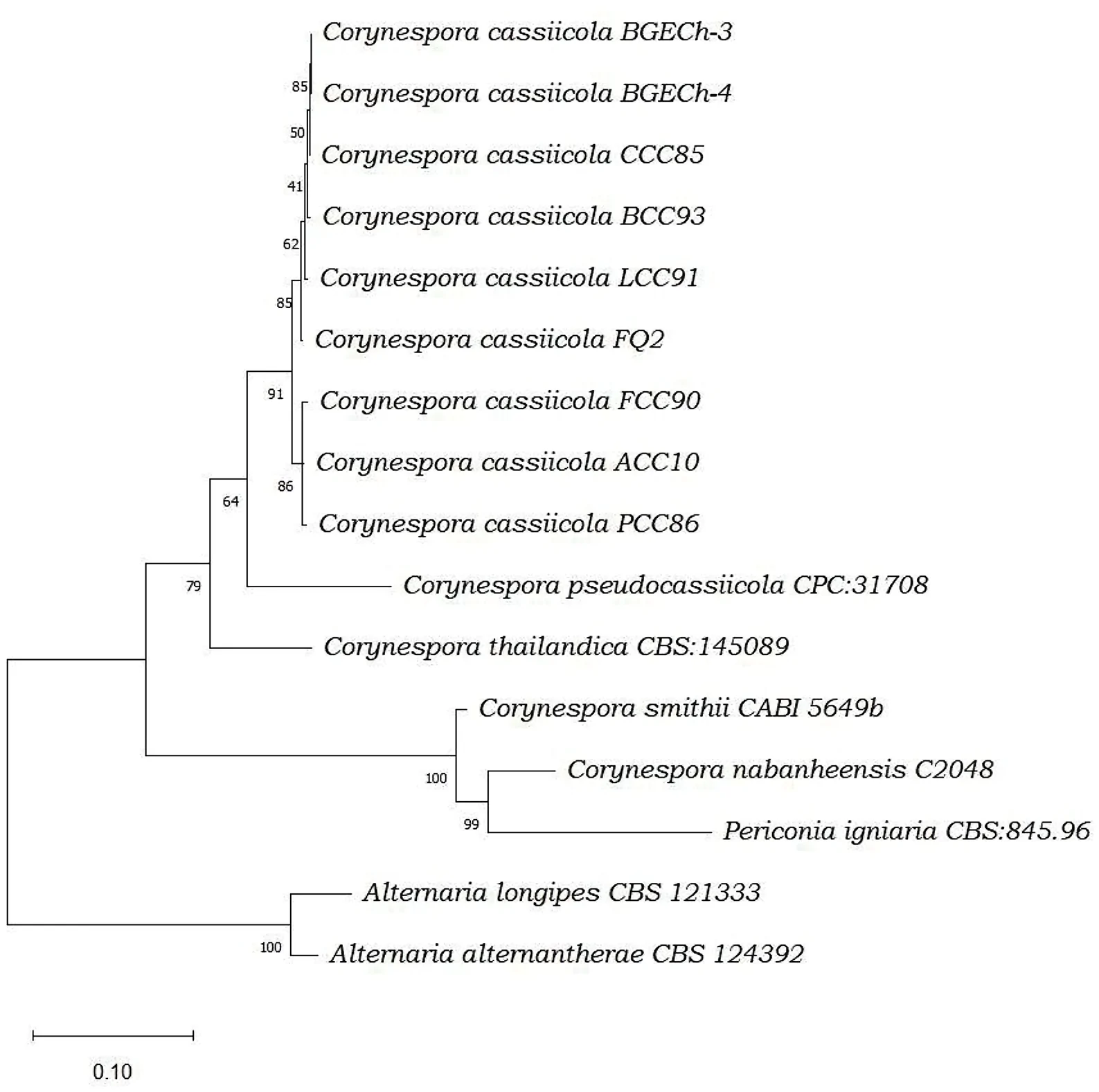
Maximum likelihood phylogenetic tree of *C. cassiicola* based on combined ITS-rDNA and *TEF1-α* gene sequences. The isolates BGECh-3 and BGECh-4from the present study clustered with the reference strain of *C. cassiicola.* Sequences generated in this study are indicated by colored blocks. Bootstrap support values (%) based on 1,000 replicates are shown at the corresponding internal nodes. The tree is drawn to scale, with branch lengths measured in the number of substitutions per site. The final dataset comprised 16 nucleotide sequences and 1060 aligned positions.

The internal transcribed spacer (ITS) region and the translation elongation factor 1-alpha (*TEF-1α*) gene are widely recognized as reliable DNA barcodes for fungal species identification [25,33]. To confirm the identity of the isolates, both ITS and *TEF-1α* regions were amplified, sequenced, and compared with reference sequences available in the NCBI GenBank database. The newly generated sequences were deposited in GenBank and assigned accession numbers PQ588139 and PQ588449 for the ITS region, and PQ998226 and PQ998227 for the *TEF-1α* gene. BLASTn analysis showed that the ITS sequences shared 100% nucleotide identity with *C. cassiicola* isolate CC-01 (GenBank accession no. KP759967), while the *TEF-1α* sequences exhibited 99.69% nucleotide similarity with isolate Cc_01 (GenBank accession no. MK882240). Furthermore, maximum likelihood phylogenetic analysis based on concatenated ITS and *TEF 1-α* sequences demonstrated that isolates BGECh-3 and BGECh-4 clustered with reference strains of *C. cassiicola*, thereby confirming their taxonomic identity (Fig 3).

### Pathogenicity assay

**Fig 4:**
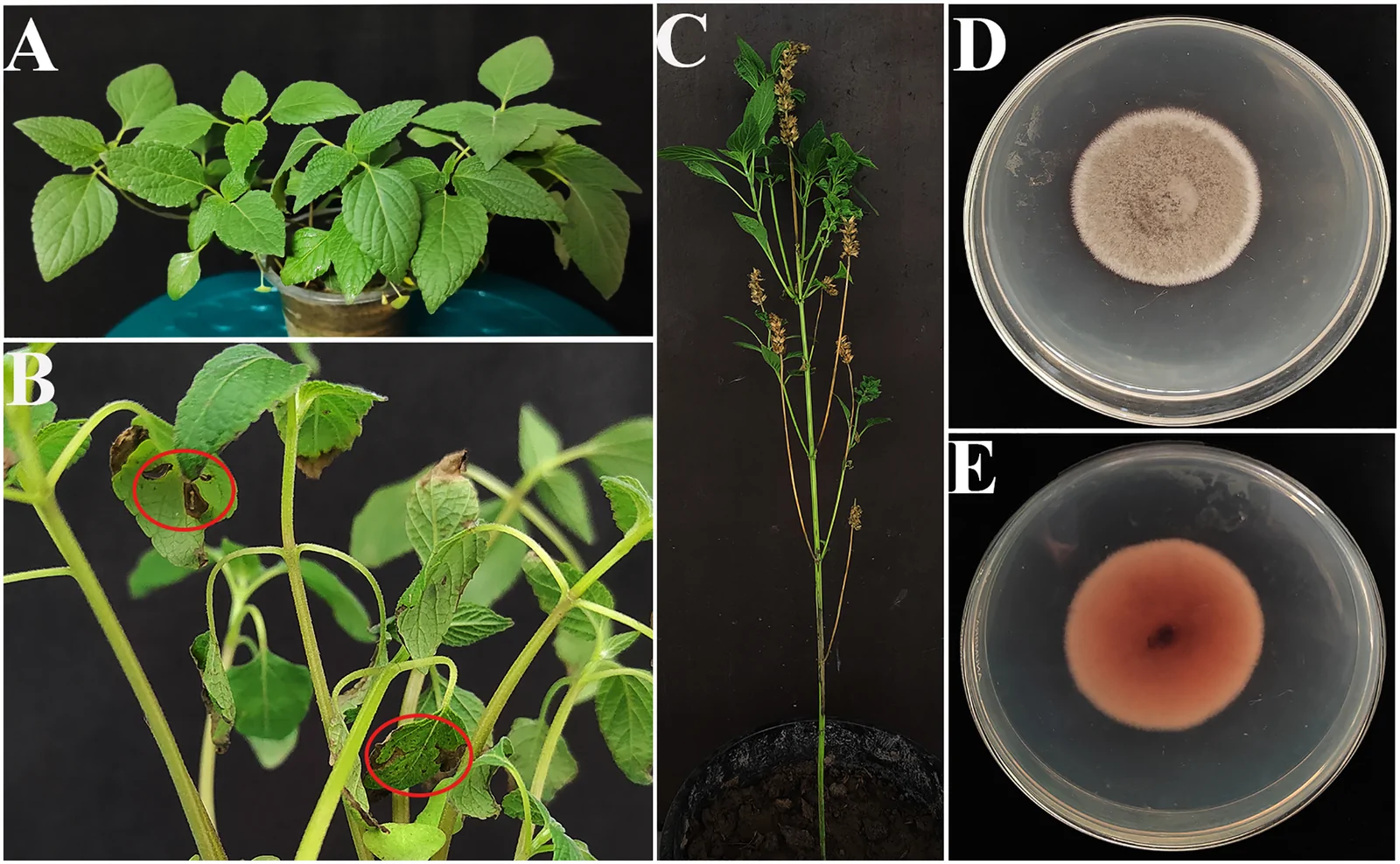
Disease symptoms on chia plant inoculated with C. cassiicola isolate BGECh-3 under controlled conditions (10-15 dpi). (A) Water treated control plants remained symptomless; (B) inoculated seedlings showing typical disease symptoms in red circle; (C) infected inflorescence; (D-E) colony morphology of reisolated pathogen recovered from the artificially inoculated plant (obverse and reverse views).

Inoculated seedlings developed typical target spot symptoms within 6–8 days, and inflorescences and stems within 10–15 days (Fig 4B,C), while controls remained symptomless (Fig 4A). The pathogen was successfully re-isolated from symptomatic tissues of all inoculated plants (Fig 4D,E) confirming *C. cassiicola* as the causal agent of target spot of chia by morphological and molecular characterization. However, no *C. cassiicola* was recovered from healthy plants. These results indicate that the isolates BGECh-3 and BGECh-4 are the causal agent of target spot disease in chia in Bangladesh.

### Host range of *C. cassiicola* isolates

**Fig 5:**
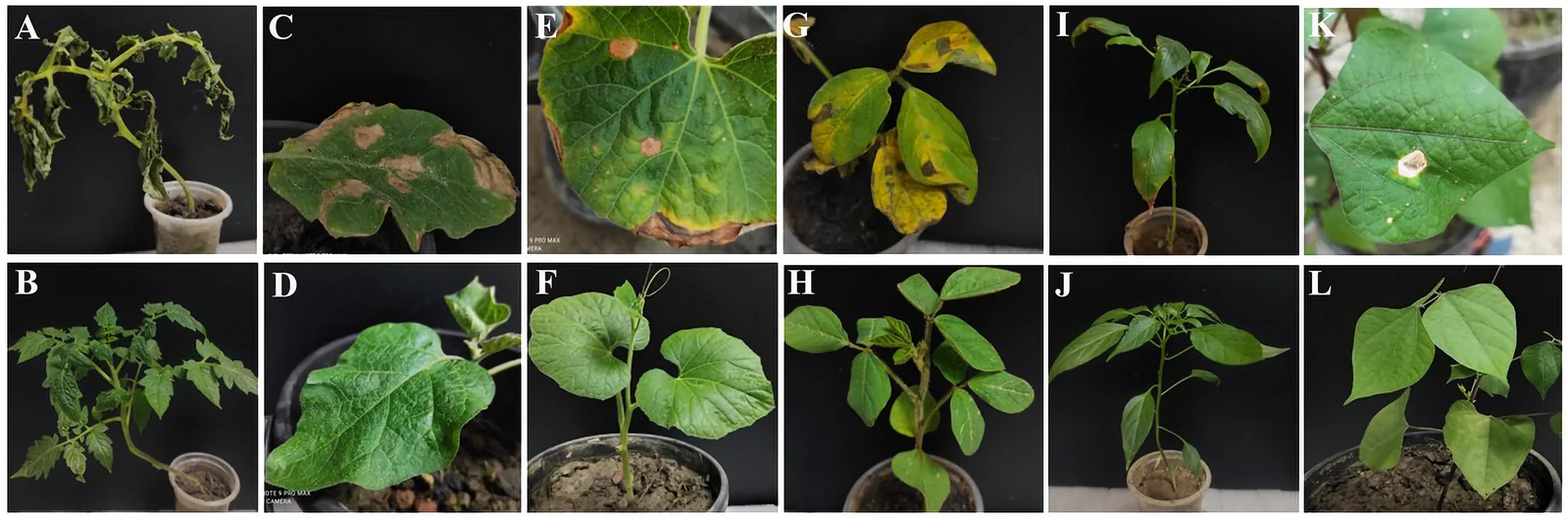
Pathogenicity assessment of *C. cassiicola* on multiple host plants under controlled conditions. Respective symptoms of target leaf spots are presented on inoculated plants(A) tomato, (C) brinjal, (E) bottle-gourd, (G) soybean, (I) chili, and (K) country-bean. Corresponding non-inoculated control plants are presented directly below each treatment: (B), (D), (F), (H), (J), and (L), respectively. Symptom development was monitored at 3-day intervals. Typical target spot symptoms, including necrotic lesions with concentric rings of varying severity, were observed in all inoculated plants, whereas no symptoms developed in the control plants.

Both the isolates were pathogenic to all tested hosts but differed in disease severity. Tomato exhibited the highest susceptibility, with severe wilting and complete plant collapse within 10 days after inoculation (Fig 5A). Eggplant and soybean also developed severe disease symptoms, whereas bottle gourd exhibited prominent necrotic leaf lesions without complete plant collapse (Fig 5C, E, G). In soybean, initial symptoms appeared 5 days after inoculation, beginning as brown discoloration on older leaves. As the disease progressed, whole plants gradually turned yellow and eventually dried (Fig 5G). In contrast, chili and country bean developed localized target spot lesions with characteristic concentric rings at12–14 days after inoculation, indicating comparatively lower susceptibility (Fig 5I, K).

### Fungicide sensitivity assays

Fungicide sensitivity was evaluated based on mycelial growth inhibition. Both *C. cassiicola* isolates showed similar sensitivity to the tested fungicides, and the data were then pooled. Mycelial growth inhibition generally increased with increasing fungicide concentration, although the magnitude of response varied among fungicides (S1 Table).

**Fig 6:**
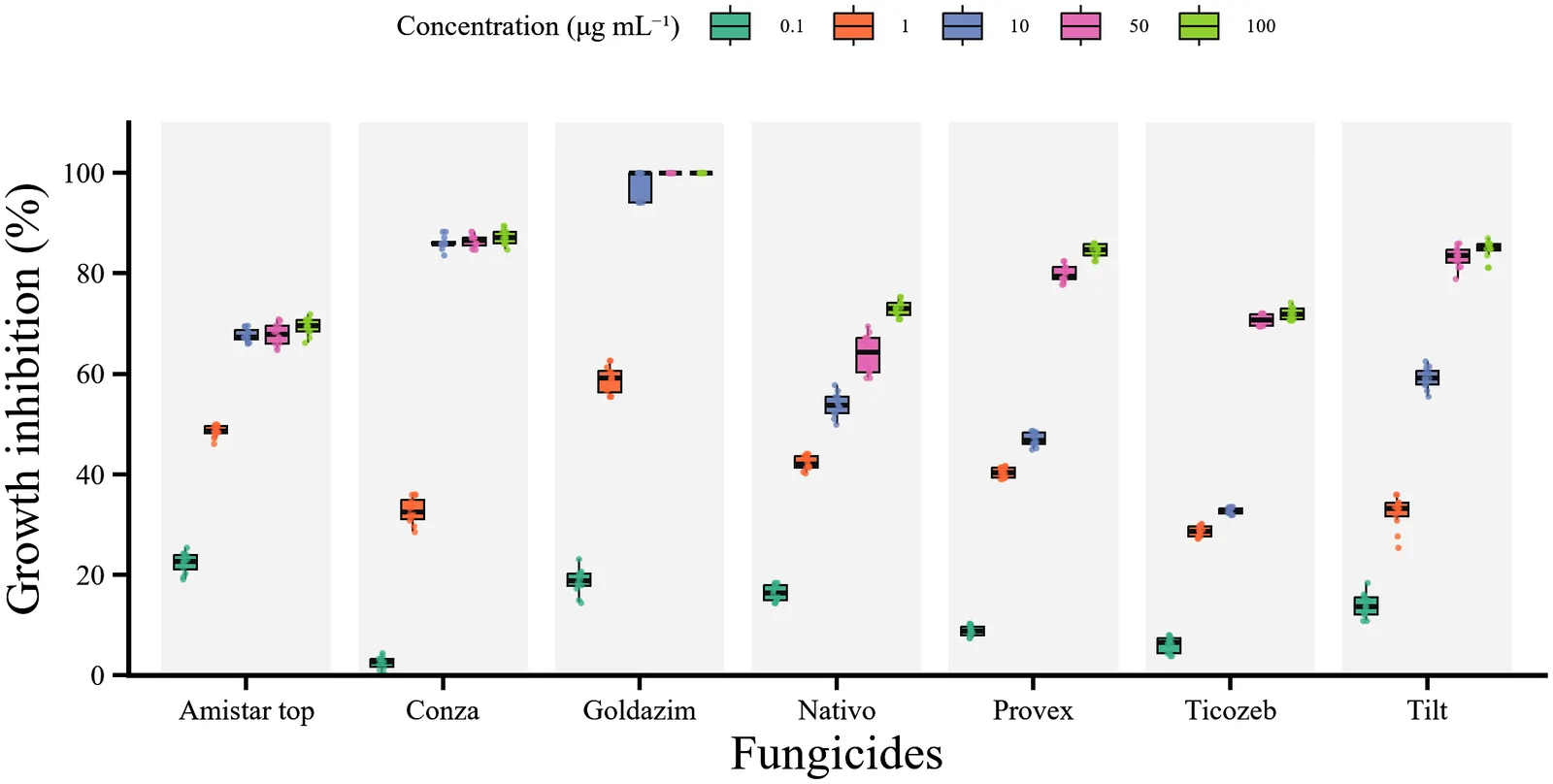
Mycelial growth inhibition (%) of C. cassiicola under four fungicides across five concentrations (0.1, 1, 10, 50, and 100 μg mL ^−^ ¹). Boxplots represent pooled inhibition rate from both *C. cassiicola* isolates (BGECh-3 and BGECh-4), based on six independent biological replicates per treatment. Overlaid points indicate individual observations. The central line denotes the median, the boxes represent the interquartile range (IQR), and the whiskers indicate the range of observed values.

**Fig 7:**
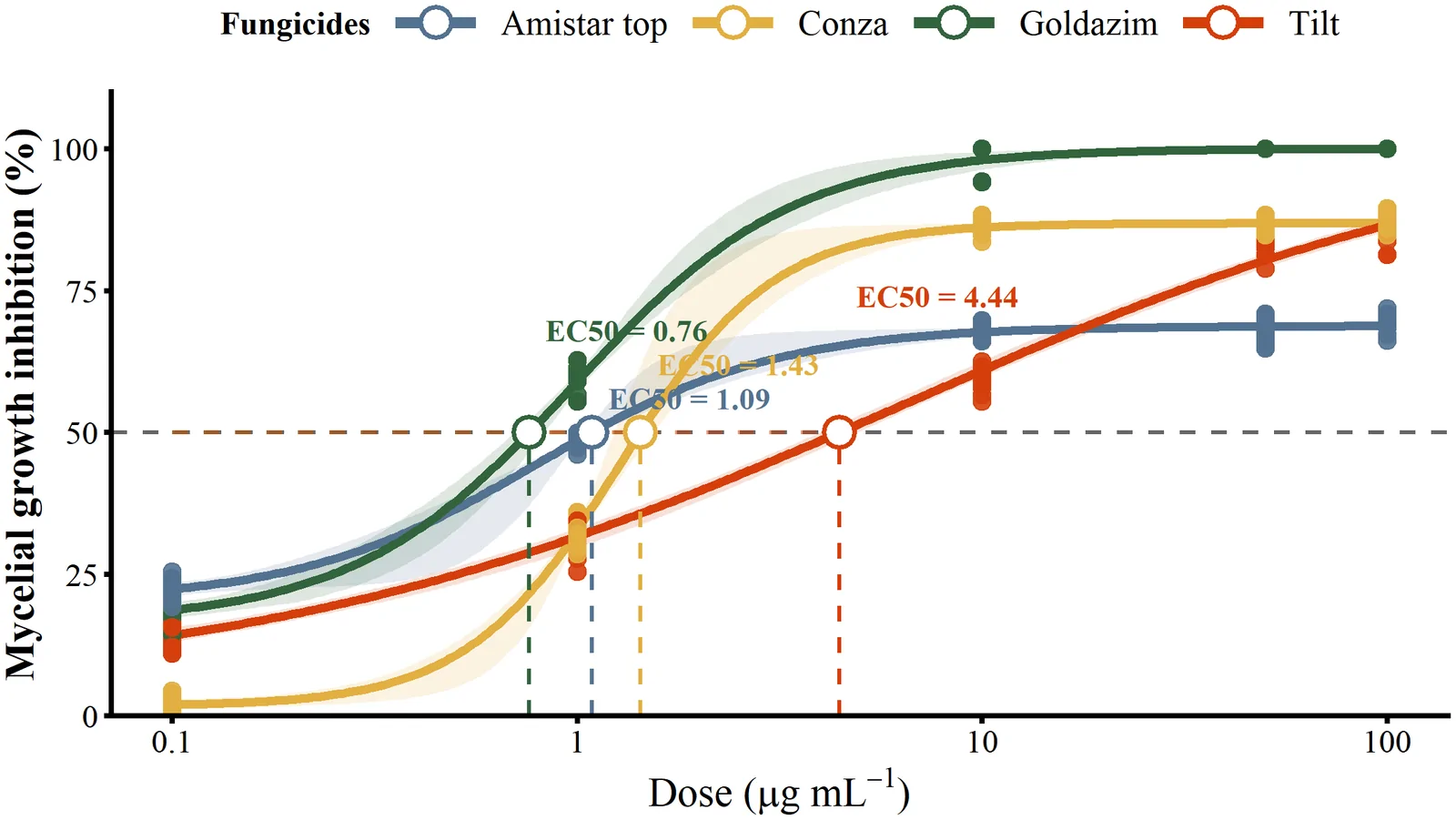
EC50 (50% mycelial inhibition) determination of fungicides against *C. cassiicola* based on mycelial growth inhibition. Dose-response curves show the relationship between fungicide concentration (μg mL ^−^ ¹) and mycelial growth inhibition for Amistar Top, Conza, Goldazim, and Tilt. Solid lines represent fitted models for each fungicide and shaded ribbons indicate 95% bootstrap confidence intervals. Dots represent observed inhibition values from individual replicates. Open circles indicate the estimated EC_50_ for each fungicide, and dashed lines denote the corresponding 50% inhibition level and EC_50_ positions.

Among the seven tested fungicides, Goldazim (carbendazim) and Conza (hexaconazole) produced the greatest inhibition of mycelial growth. At 10 μg mL ^−^ ¹, Goldazim achieved 98.05 ± 2.88% inhibition and completely suppressed at higher concentrations (50–100 μg mL ^−^ ¹). Conza reduced mycelial growth by 86.17 ± 1.31% at 10 μg mL ^−^ ¹, with only slight increases to 86.46 ± 1,28% and 87.34 ± 1.49% at 50 and 100 μg mL ^−^ ¹, respectively, indicating that increasing the concentration beyond 10 μg mL ^−^ ¹ provided little additional inhibition under the conditions tested. Amistar top (azoxystrobin + difenoconazole) and Tilt (propiconazole) exhibited intermediate activity, with inhibition ranging from 67.77 ± 1.34% to 69.52 ± 1.66% and 59.30 ± 2.08% to 84.81 ± 1.87%, respectively, across the tested concentrations. Nativo (tebuconazole + trifloxystrobin) and Provax (carboxin + thiram) showed moderate efficacy, reaching 72.93 ± 1.65% and 84.62 ± 1.30% inhibition at 100 μg mL ^−^ ¹, respectively. In contrast Tricozeb (mancozeb) was the least effective fungicide, producing only 32.81 ± 0.64% inhibition at 10 μg mL ^−^ ¹ and 69.04 ± 1.83 inhibition at the higher concentration tested (Fig 6) (Fig 7).

Similar trends were observed in the dose-response analysis (Fig 7; S2 Table). Four-parameter log-logistic models provided reliable EC_50_ estimates for Goldazim, Amistar Top, Conza, and Tilt, demonstrating concentration-dependent inhibition. Goldazim exhibited the lowest EC_50_ (0.76 μg mL ^−^ ¹), followed by Amistar Top (1.09 μg mL ^−^ ¹) and Conza (1.43 μg mL ^−^ ¹), whereas Tilt showed a comparatively higher EC_50_ (4.44 μg mL ^−^ ¹), indicating lower fungicidal activity under tested conditions. Reliable EC_50_ estimates could not be obtained for Nativo, Provax, and Ticozeb because the fitted model showed poor precision and wide confidence intervals (S2 Table). *C. cassiicola*

## Discussion

This study for the first time demonstrated the occurrence of target spot disease on chia caused by *C. cassiicola* in Bangladesh. The disease symptoms observed in the present study were typical of Corynespora target spot reported in many other crops [13,34]. The emergence of this pathogen on chia is of particular significance because chia is gaining importance as a high-value food crop in the region. Apart from Bangladesh, this disease has only been reported on chia in India, where the disease was first observed during a field outbreak in Karnataka in 2020 [34]. This suggests that the pathogen may be expanding its geographic distribution on chia within South Asia. In Bangladesh, *C. cassiicola* has previously been reported on okra [35], whereas the present study represents its first occurrence on chia, indicating a potential host shift or adaptation that could pose new risks to crop production. *C. cassiicola*

Morphological characterization of the pathogen supported its preliminary identification as *Corynespora sp*. The isolates in this study produced conidia of variable size and shape, occasionally forming short chains and containing multiple pseudosepta, most commonly 2–5, which is consistent with the known morphological features of the genus *Corynespora* [15,32,36]. However, a predominance of cylindrical and straight conidia was observed in our study, similar to those previously described for this pathogen isolated from soybean [13], whereas some other studies have reported predominantly obclavate and curved conidia in culture media [16,37]. However, the uniformity of conidial morphology among the isolates suggested that a single species was responsible for the disease outbreak on chia in the study area.

The integration of molecular and morphological analysis provides a more reliable approach for fungal species identification, particularly in fungi exhibiting cryptic morphology [38]. The internal transcribed spacer (ITS) region and the translation elongation factor 1-alpha *(TEF-1α)* gene are widely used as molecular barcodes for fungal species identification [34,39,40]. Although the ITS region is effective for genus-level identification, it may lack sufficient variability to distinguish closely related species within certain genera [41,42]. Therefore, sequencing two or more loci provides more reliable and comprehensive species identification. The *TEF-1α* gene has high phylogenetic resolution and is particularly useful for differentiating cryptic species within a genus [43,44]. Phylogenetic analysis based on combined ITS and *TEF-1α* sequences of two representative isolates showed that the isolates clustered with reference strains of *C. cassiicola*. This multilocus approach provided strong molecular evidence and further confirmed the identity of the pathogen.

Pathogenicity tests were performed to determine the virulence of the *C. cassiicola* isolates, and the results clearly demonstrated their ability to infect chia and reproduce the characteristic target spot symptoms observed under field conditions. The inoculated plants developed typical concentric necrotic lesions, thereby fulfilling Koch’s postulates and confirming the causal role of the pathogen. *C. cassiicola* is widely recognized as a destructive and highly adaptable pathogen, reported to cause leaf spot, defoliation, and inflorescence blight in more than 400 plant species worldwide [12]. Its broad host range and aggressive infection strategy make it particularly concerning in diversified cropping systems.

In the present study, *C. cassiicola* was identified for the first time as the causal agent of target spot disease of chia in Bangladesh, highlighting the emergence of a new host–pathogen association in the country. Importantly, cross-pathogenicity assays revealed that the isolates were capable of infecting other economically important vegetable crops cultivated in Bangladesh (Fig 5). This finding suggests that the pathogen possesses a flexible host adaptation capacity, increasing the risk of spillover to additional crops. Given Bangladesh’s intensive and often overlapping vegetable production systems, such cross-infectivity may facilitate rapid dissemination across fields and seasons [45]. If not properly managed, the pathogen could establish itself in multiple hosts, creating a persistent inoculum reservoir and escalating disease pressure. Therefore, the emergence of *C. cassiicola* on chia should be viewed not as an isolated incident but as a potential threat to a wider range of vegetable crops in Bangladesh, warranting immediate surveillance and integrated disease management strategies to prevent further spread and host expansion.

The management of target spot disease largely relies on chemical control, particularly under environmental conditions that favor rapid disease development. In the present study, seven fungicides with different modes of action were evaluated in vitro to determine their effectiveness against the pathogen. Among them, Goldazim had a lower EC_50_ value (0.76 μg mL ^−^ ¹) and complete suppression was achieved at 50 and 100 μg mL ^−^ ¹, indicating high antifungal activity. This EC_50_ value is consistent with previous reports of carbendazim-sensitive *C. cassiicola* population [46]. The superior performance of the fungicide suggests that this could be a promising candidate for the management of target spot disease of chia under field conditions. Goldazim contains carbendazim, a systemic benzimidazole fungicide, known for its effectiveness against a wide range of fungal pathogens, including *C. cassiicola*. Carbendazim suppresses fungal growth by binding to β-tubulin and disrupting microtubule assembly during mitosis, thereby inhibiting fungal cell division, which likely accounts for its high efficacy [47]. However, carbendazim-resistant *C. cassiicola* populations have been documented in multiple cucumber-growing provinces of China, highlighting the regional risk of fungicide resistance and emphasizing the importance of resistance monitoring and judicious fungicide use [20,24]. *C. cassiicola*

The response of *C. cassiicola* to the Conza and Amistar Top indicates a clear distinction between potency and maximum efficacy. The mixture of azoxystrobin + difenoconazole (Amistar Top) showed a lower EC₅₀, suggesting greater activity at low concentrations, but resulted in lower inhibition at 10 µg ml ^−^ ¹ compared with hexaconazole (Conza). This suggests that the mixture is more potent but has a limited maximum inhibitory effect. In contrast, hexaconazole achieved greater inhibition at higher concentration (10 µg ml ^−^ ¹), indicating stronger overall efficacy. These differences may be attributed to their distinct modes of action. Azoxystrobin, a QoI fungicide, inhibits mitochondrial respiration by blocking electron transport, while difenoconazole and hexaconazole, both triazoles, interfere with ergosterol biosynthesis [47–49]. The combined action of azoxystrobin and difenoconazole in the mixture may result in strong initial growth suppression; however, the reduced efficacy observed at higher concentrations may reflect differences in the intrinsic sensitivity of *C. cassiicola* to these active ingredients or other physiological factors, although this requires further investigation. Conversely, hexaconazole may exert a more sustained inhibitory effect on membrane integrity at higher concentrations, leading to greater overall growth suppression. Interestingly, only a few studies have evaluated the hexaconazole against *C. cassiicola*, and those studies reported effective disease control under both in vitro and field conditions [50,51].Additionally, differences in fungistatic versus fungicidal activity, uptake efficiency, and detoxification mechanisms in the pathogen may further contribute to the observed response. Therefore, EC₅₀ values alone may not fully represent fungicide performance against *C. cassiicola*.

However, it is important to note that reliance solely on fungicides may not be sustainable in the long term due to the risk of fungicide resistance development in *C. cassiicola*. This pathogen highly adaptable and genetically diverse and is classified by FRAC as “high risk” for developing fungicide resistance [12]. Therefore, integrated disease management strategies, including crop sanitation, use of disease-free planting material, monitoring of environmental conditions, rotation with non-host crops, and rational fungicide rotation, should be considered to minimize disease pressure and prolong fungicide efficacy. Given the broad host range of *C. cassiicola*, rotation with susceptible crops may provide limited benefits because the pathogen can persist on alternative hosts. Further field-based evaluations are necessary to validate the in vitro findings and to optimize application timing and dosage for effective disease control.

## Conclusion

This study reports, for the first time, the occurrence of *C. cassiicola*, the causal agent of target spot disease in chia, in Bangladesh. The findings highlight the importance of continuous surveillance of emerging diseases in newly introduced or expanding crops. Monitoring the host range of the pathogen through field surveys will also be important for designing effective crop rotation strategies and crop isolation practices to limit disease spread. The fungicide sensitivity assay further demonstrated the effectiveness of three fungicides Goldazim, Conza and Amistar Top in suppressing the growth of *C. cassiicola*. Nevertheless, the widespread use of carbendazim based fungicides, together with documented occurrence of carbendazim resistance in *C. cassiicola* populations from other cropping systems, underscore the need for periodic monitoring of fungicide sensitivity in chia growing regions to detect shifts in pathogen susceptibility, preserve fungicide efficacy, and guide evidence-based resistance management. Given the increasing cultivation and economic importance of chia, early detection and effective management of target spot disease will be critical to prevent potential yield losses and ensure sustainable crop production. Future research focusing on pathogen diversity, epidemiology, fungicide resistance, and integrated disease management strategies will further improve our understanding and help develop effective control measures for this emerging disease.

## Supporting information

S1 Table Analysis of variance of fungicide treatment and dose effects on mycelial growth inhibition of *C. cassiicola.* The analysis evaluated the main effects of fungicide treatment and dose, as well as their interaction, on mycelial growth inhibition (%).
(DOCX)

S2 Table Estimated EC_50_ with 95% Confidence intervals for the tested fungicides against *C. cassiicola.* EC_50_ values were estimated by fitting a four-parameter log- logistic (LL.4) dose–response model using the drc package in R. The 95% confidence intervals were obtained using 1,000 bootstrap iterations. “Reliable” indicates EC_50_ estimates with positive confidence interval limits and a standard error smaller than the estimated EC₅₀ value.
(DOCX)
