# Supplementary material for: First occurrence of Corynespora cassiicola infecting chia plant in Bangladesh and sensitivity of the pathogen to selected fungicides
Source: PLoS One. 2026 Sep 17;21(9):e0349471. doi: 10.1371/journal.pone.0349471 (PMC13585262; doi:10.1371/journal.pone.0349471)
Supplement: Supplementary file 1 [file pone.0349471.s001.docx]

**Supporting Information**

**S1 Table. Analysis of variance of fungicide treatment and dose effects on mycelial growth inhibition of *C. cassiicola*.** The analysis evaluated the main effects of fungicide treatment and dose, as well as their interaction, on mycelial growth inhibition (%).

|  | **Sum Sq** | **Df** | **F value** | **Pr(>F)** |
| --- | --- | --- | --- | --- |
| (Intercept) | 1287263.83 | 1 | 377231.205976 | 0.000000e+00 |
| Treatment | 37249.175 | 6 | 1812.3468 | 1.074940e-278 |
| Dose (ppm) | 281619.665 | 4 | 18313.0951 | 0.000000e+00 |
| Treatment: Dose (ppm) | 30853.383 | 24 | 375.2904 | 2.631677e-250 |
