# Supplementary material for: First occurrence of Corynespora cassiicola infecting chia plant in Bangladesh and sensitivity of the pathogen to selected fungicides
Source: PLoS One. 2026 Sep 17;21(9):e0349471. doi: 10.1371/journal.pone.0349471 (PMC13585262; doi:10.1371/journal.pone.0349471)
Supplement: Supplementary file 2 [file pone.0349471.s002.docx]

**Supporting Information**

**S2 Table. Estimated EC_50_ with 95% Confidence intervals** **for the tested fungicides against *C. cassiicola*.** EC₅₀ values were estimated by fitting a four-parameter log- logistic (LL.4) dose–response model using the drc package in R. The 95% confidence intervals were obtained using 1,000 bootstrap iterations. “Reliable” indicates EC₅₀ estimates with positive confidence interval limits and a standard error smaller than the estimated EC₅₀ value.

| **Treatment** | **Estimate** | **Std.Error** | **Lower** | **Upper** | **Reliable** |
| --- | --- | --- | --- | --- | --- |
| Amistar top | 1.085132 | 0.04214988 | 1.0006955 | 1.1695682 | **Yes** |
| Conza | 1.433674 | 0.08139766 | 1.2706153 | 1.5967335 | **Yes** |
| Goldazim | 0.759700 | 0.03518529 | 0.689054 | 0.8300232 | **Yes** |
| Nativo | 4.034224 | 3.27620243 | -2.5287977 | 10.5972465 | No |
| Provex | 5.236074 | 10.42988039 | -15.6574874 | 26.1296347 | No |
| Ticozeb | 19.0627838 | 19.80051212 | -20.602408 | 58.7279760 | No |
| Tilt | 4.4446835 | 0.66673242 | 3.109058 | 5.7803090 | **Yes** |
